# Comment on “Laparoscopic Paraesophageal Hernia Repair: to Mesh or Not to Mesh”

**DOI:** 10.1097/AS9.0000000000000304

**Published:** 2023-06-28

**Authors:** Alberto Aiolfi, Davide Bona, Gianluca Bonitta, Michele Manara, Luigi Bonavina

**Affiliations:** From the I.R.C.C.S. Ospedale Galeazzi – Sant’Ambrogio, Division of General Surgery, Department of Biomedical Science for Health, University of Milan, Milan, Italy.

We read the systematic review and meta-analysis by Angeramo and Schlottmann.^1^ This study aimed to evaluate the effect of mesh augmented cruroplasty versus simple cruroplasty in terms of hiatus hernia recurrence prevention after laparoscopic paraesophageal hernia repair. The article questioned the utilization of mesh-reinforced cruroplasty in the early- and medium-term follow-up since similar recurrence risk ratios (RR) were found between groups.

We thank the authors for their contribution and attempt to clarify such an intriguing topic. However, we believe that there has been some data misinterpretation possibly leading to misunderstanding of outcomes. When assessing the RR for early (<6 months) and late (>6 months) recurrence, we found some incongruities between the data presented in the Forrest plot and the results reported in selected trials. This occurred because recurrence was not uniformly defined among trials and because the authors included patients lost at follow-up. This conceivably introduced an overestimation of the causative effect. Moreover, the related heterogeneity was significant for both early (*I*^2^ = 40%) and late (*I*^2^ = 64%) recurrence while the leave-one-out sensitivity analysis did not reveal any significant changes in the overall effect. Diverse inclusion criteria, hernia size, surgical indications, and definition of recurrence were heterogeneous, while surgeon experience, mesh materials, shape, crural fixation, and type of fundoplication were assessed thus possibly explaining such a noteworthy interstudy heterogeneity. All these issues significantly limit the validity and robustness of the study, and suggest more pondered conclusions.

Given these methodological inconsistencies, we performed a new pairwise analysis focusing on early recurrence (≤6 months). We performed random effect frequentist meta-analysis using the Inverse variance method, the restricted maximum-likelihood estimator for the between study variance (τ2) and the Q-Profile method for confidence intervals. We considered a more homogeneous definition of recurrence among trials (both anatomic and symptomatic) and excluded patients lost to follow-up (Fig. 1). Similar to the analysis of Angeramo and Schlottmann,^1^ we found no differences for mesh versus no mesh comparisons in terms of hiatus hernia recurrence (RR = 0.55; 95% Confidence Interval [CI] = 0.19–1.56; *P* = 0.26) and in heterogeneity (*I*^2^ = 40%; 95% CI = 0.0%–78%; *P* = 0.15). However, the one-leave-out sensitivity analysis showed that after exclusion of the study by Oor et al, the heterogeneity decreased to zero (*I*^2^ = 0.0%) (Supplemental Figure 1, http://links.lww.com/AOSO/A227). Remarkably, the pairwise analysis performed after the exclusion of the study by Oor et al showed a significantly reduced RR of recurrence for mesh-reinforced cruroplasty versus simple repair (RR = 0.35; 95% CI = 0.14–0.83; *P* = 0.02; *I*^2^ = 0.0%) (Fig. 2). Therefore, this heterogeneity-zero pairwise analysis seems to support the utilization of mesh for crural augmentation.

**FIGURE 1.**
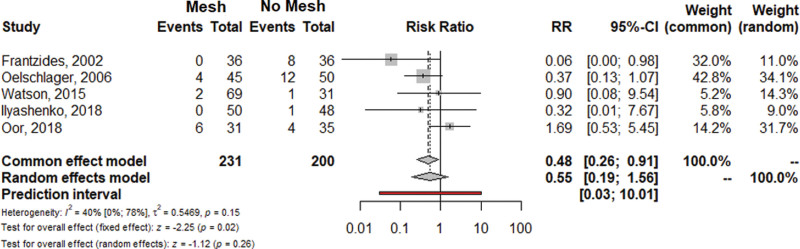
Forrest plot for hiatus hernia recurrence. The analysis was performed using the R software program, version 3.2.2. 95% CI indicates confidence interval; RR: risk ratio.

**FIGURE 2.**
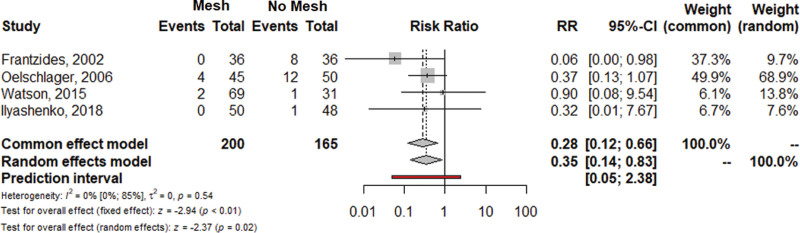
Forrest plot for hiatus hernia recurrence after the exclusion of the study by Oor et al. The analysis was performed using the R software program, version 3.2.2. 95% CI indicates confidence interval; RR: risk ratio.

The repair of the hiatus is a matter of intense discussion while the “ideal” method for crural repair is debated since the first description by Soresi.^3,4^ Our data support previous studies that conveyed reduced risk for recurrence after mesh reinforced cruroplasty.^5,6^ At present, this topic remains open to debate and consensus is limited due to the complexity of factors involved in paraesophageal hernia recurrence and the lack of standardization of current surgical techniques.^7^ We believe that, in selected patients, use of a resorbable synthetic mesh as an adjunct to adequate distal esophageal dissection, standard cruroplasty, fundic mobilization, and posterior partial fundoplication has the potential to reduce early recurrence rates and improve quality of life outcomes.^8,9^

## Supplementary Material


