# Supplementary material for: Comment on “Laparoscopic Paraesophageal Hernia Repair: to Mesh or Not to Mesh”
Source: Ann Surg Open. 2023 Jun 28;4(3):e304. doi: 10.1097/AS9.0000000000000304 (PMC10513235; doi:10.1097/AS9.0000000000000304)
Supplement: Supplementary file 1 [file as9-4-e304-s001.pdf]

```
> metainf(m1,pooled="random")
Influential analysis (Random effects model)

                                RR            95%-CI p-value   tau^2    tau    I^2
Omitting Frantzides, 2002    0.7247 [0.2667; 1.9692]  0.5278  0.3547  0.5955  22.2%
Omitting Oelschlager, 2006   0.6030 [0.1384; 2.6261]  0.5004  0.9703  0.9851  41.6%
Omitting Watson, 2015        0.4718 [0.1277; 1.7429]  0.2599  0.9048  0.9512  54.5%
Omitting Ilyashenko, 2018    0.5667 [0.1746; 1.8398]  0.3445  0.7054  0.8399  54.1%
Omitting Oor, 2018           0.3461 [0.1439; 0.8324]  0.0178  0.0000  0.0000   0.0%

Pooled estimate              0.5488 [0.1925; 1.5647]  0.2617  0.5469  0.7395  40.3%

Details on meta-analytical method:
- Mantel-Haenszel method
- Restricted maximum-likelihood estimator for tau^2
```
